# Long-Term Outcomes of Mechanical Thrombectomy for Stroke: A Meta-Analysis

**DOI:** 10.1155/2019/7403104

**Published:** 2019-05-02

**Authors:** David J. McCarthy, Anthony Diaz, Dallas L. Sheinberg, Brian Snelling, Evan M. Luther, Stephanie H. Chen, Dileep R. Yavagal, Eric C. Peterson, Robert M. Starke

**Affiliations:** ^1^Department of Neurological Surgery, University of Miami Miller School of Medicine, Miami, FL 33136, USA; ^2^Department of Neurology, University of Miami Miller School of Medicine, Miami, FL 33136, USA; ^3^Department of Neurological Surgery and Radiology, University of Miami Miller School of Medicine, Miami, FL 33136, USA

## Abstract

Mechanical thrombectomy (MT) has become the standard treatment for large vessel occlusion (LVO) in acute ischemic stroke (AIS). Few studies have investigated long-term outcomes for AIS treated with MT. Therefore, a pooled meta-analysis using data from randomized clinical trials (RCT) was performed to assess for long-term clinical outcomes. A systematic literature search was conducted on 27 September 2017, by searching the English literature in the Cochrane Library, MEDLINE, and Embase for RCTs investigating long-term outcomes (greater than standard 3-month timepoint) of endovascular intervention versus medical management for patients with AIS. The study was carried out according to PRISMA guidelines and random effects analysis was carried out to account for heterogeneity. Three trials were included: IMS III, MR CLEAN, and REVASCAT, comprising a total of 1,362 patients. Long-term clinical outcomes were available for 1-year follow-up in IMS III and REVASCAT and at 2 years in MR CLEAN. Functional independence at long-term follow-up favored endovascular stroke intervention (OR 1.51;* p =* 0.02). When stratified by LVO inclusion criteria, greater endovascular functional independence benefits were observed (OR 1.85;* p* = 0.0005). There was a significant difference between the 2 arms in favor of endovascular therapy for the quality of life at long-term follow-up (mean difference 0.11;* p* = 0.0002). No difference in mortality at long-term follow-up was observed (OR 0.82;* p* = 0.12). We conclude that endovascular therapy results in favorable outcomes at long-term follow-up for patients with acute ischemic stroke compared to standard medical treatment alone and that the 90-day timepoint offers a fair representation of the long-term outcomes.

## 1. Introduction

In the United States, approximately 795,000 patients suffer from an acute ischemic stroke (AIS) every year [1]. Standard of care for AIS has radically changed since the establishment of mechanical thrombectomy (MT) as an effective treatment modality. Despite poor revascularization rates, a short efficacy window, and risk of hemorrhage, intravenous thrombolysis using recombinant tissue plasminogen activator (IV-tPA) was considered the standard of care for AIS prior to the advent of MT [2–9].

The first three major randomized control trials (RCTs), comparing endovascular therapy to standard intravenous therapy, IMS III, SYNTHESIS Expansion, and MR RESCUE, reported no difference in clinical outcomes between the two treatment methods [10–12]. These studies were limited because they lacked large vessel occlusion (LVO) selection criteria and did not use stent retrievers in the endovascular therapy arm (2% IMS III, 13% SYNTHESIS Expansion and 0% MR RESCUE) [13]. However, these limitations were addressed in five succeeding randomized clinical trials (MR CLEAN, ESCAPE, EXTEND-IA, SWIFT PRIME, and REVASCAT) which subsequently displayed significant clinical improvements in both recanalization rates and clinical outcomes when comparing endovascular treatment to medical therapy alone [14–18]. As a result, MT has now been recognized as standard of care for anterior large vessel occlusions resulting in AIS [19–21]. Traditionally, stroke RCTs report clinical outcomes at a standard three-month time point [22–24]. Data from RCTs evaluating other pathologies have demonstrated discord in clinical outcomes between long-term and standard time points [25]. However, very few studies have investigated long-term outcomes for AIS treated with MT. These patients often have significant comorbidities and disability and it remains unclear whether there is a long-term benefit and if long-term follow-up is necessary.

The aim of this meta-analysis is to identify RCTs that assess long-term functional independence, mortality, and quality of life between endovascular and medical management for AIS. To determine the demand of a long-term follow-up timepoint in stroke trials, we will compare the long-term outcomes to those reported at the standard 90-day timepoint for stroke. We hypothesize that the benefits previously demonstrated at the 3-month time points will be sustained in the long-term.

## 2. Material and Methods

This study follows the guidelines set forth by the Preferred Reporting Items for Systematic Reviews and Meta-Analyses (PRISMA) statement. Prior to literature search, the meta-analysis was prospectively approved and registered in the Prospero database (ID: CRD42017077919).

### 2.1. Inclusion Criteria

The inclusion criteria for this meta-analysis are any RCT that investigates the long-term outcomes (greater than standard 3-month timepoint) of endovascular intervention versus medical management for patients with AIS.

### 2.2. Literature Search and Selection

**Figure 1:**
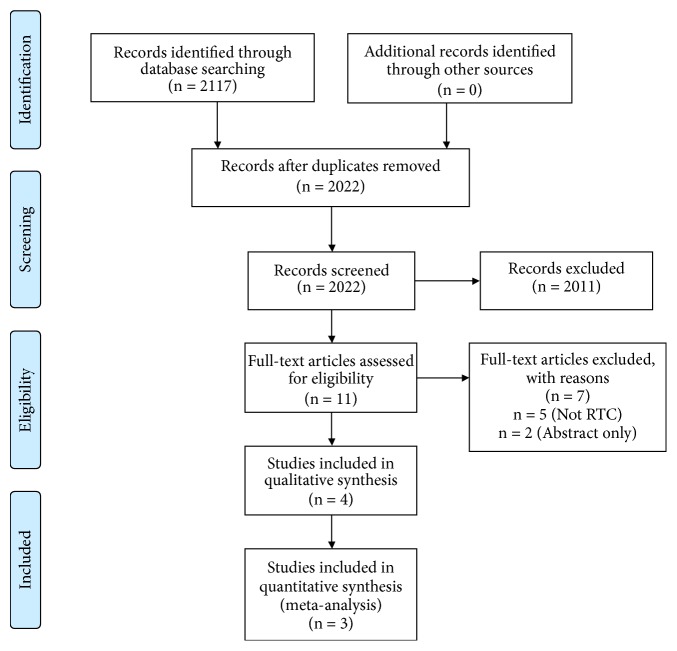
PRISMA flowchart of literature review.

A systematic literature search was conducted by searching the English literature in the Cochrane Library, Pubmed, and Embase. The following combination of MeSH terms and free text words were searched: “Cerebrovascular Stroke” or “stroke” or “Cerebrovascular Accident” or “CVA” or “stroke/surgery” and “Thrombectomy” or “Thrombectomies” or “Endovascular Procedure” or “Endovascular treatment” or “Endovascular therapy” or “Endovascular Procedures” or “Thrombectomy” and “Follow-up” or Follow-up” or “Followup” or “outcome” or “Follow-up Studies” or “Treatment Outcome” and “long-term” or “long-term.” A total of 2117 published abstracts or manuscripts were identified. Through the PRISMA flowchart (Figure 1) and the inclusion criteria, these were narrowed down to four papers for qualitative analysis, three of which were included in the qualitative analysis.

### 2.3. Data Extraction

The included study's demographic, baseline clinical, and radiographic variables were extracted. This included study trial period, inclusion/exclusion criteria, and number and location of centers that contributed. Additionally, patient age and presenting NIH Stroke Scale (NIHSS) were included. Procedural details extracted included general anesthesia usage, thrombectomy device details, and IV-tPA administration. Outcome data included functional independence using a modified Rankin Scale (mRS) score of ≤2 at >90 days after stroke, mortality rates, and symptomatic intracranial hemorrhage (sICH) rates.

### 2.4. Statistical Analysis

**Figure 2:**
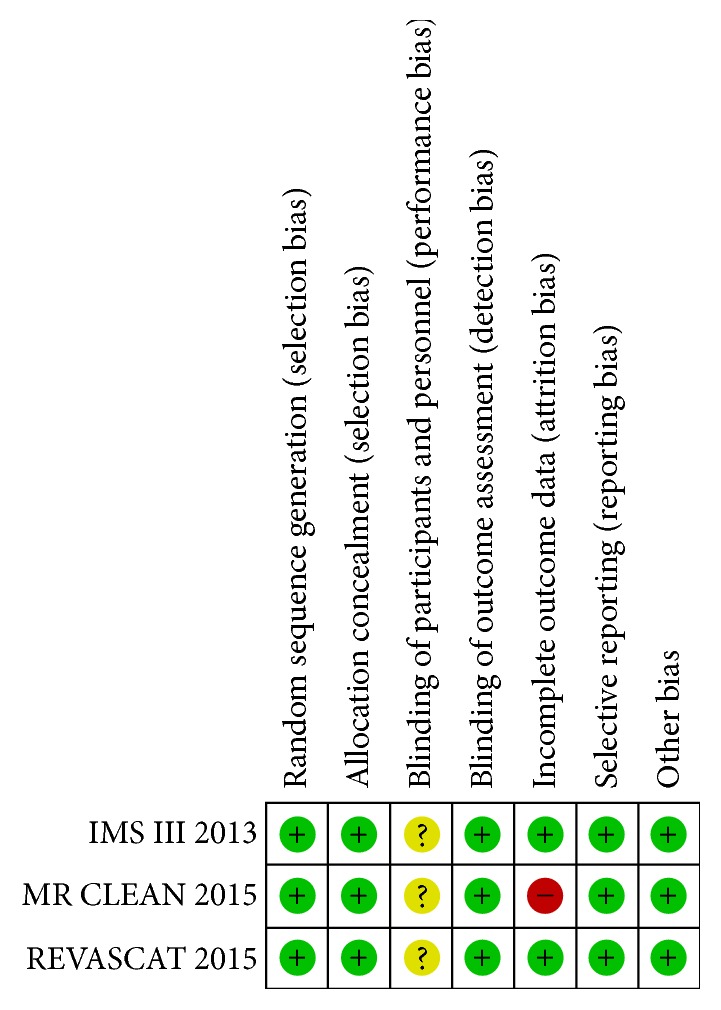
Risk assessment by of bias for included studies.

Descriptive statistics were analyzed with SAS version 9.4 (Cary, NC). The pooled data analysis was done with Review Manager version 5.3 (Copenhagen: The Nordic Cochrane Centre, The Cochrane Collaboration). Odds ratios (OR) for the studies were calculated with Mantel-Haenszel test. A random effects model was utilized, versus fixed-effects model, to account for sampling and inclusion variation between studies. Using *χ*^2^ and I^2^ test statistics, we checked for study homogeneity, with significant heterogeneity determined when both *χ*^2^ was 10% significant and I^2^ was larger than 50%. Statistical tests were two-sided, and* p* < 0.05 was considered significant. Assessment of study bias was performed using Cochrane guidelines (Figure 2). A shift analysis was carried out for the pooled mRS data using the van Elteren test variation of the Cochran-Mantel-Haenszel test [26]. The statistical program used was SAS 9.4 (Cary, NC).

### 2.5. Data Availability Statement

All relevant data used in this article is available within the article results section.

## 3. Results

### 3.1. Study Selection

The search generated three multicenter, prospective RCTs: Interventional Management of Stroke (IMS) III, Multicenter Randomized Clinical Trial of Endovascular Treatment for Acute Ischemic Stroke in the Netherlands (MR CLEAN), and Endovascular Revascularization With Solitaire Device Versus Best Medical Therapy in Anterior Circulation Stroke Within 8 Hours (REVASCAT), comprising a total of 1,362 patients for inclusion in the meta-analysis [27–29]. The three trials were reviewed for risks of bias, and all demonstrated low risk for selection, detection, attrition, and reporting biases (Figure 2). Though, treatment teams and participants were not blinded leaving the trials susceptible to performance bias. IMS III and REVASCAT both had early study termination.

### 3.2. Demographics and Study Characteristics

Key differences of the included trials are summarized in Table 1. IMS III was published in 2013 while MR CLEAN and REVASCAT were published in 2015. The total number of participating centers was 78 (range 5-58 centers). Long-term clinical outcomes were assessed at 1-year follow-up in both IMS III and REVASCAT and at 2 years in MR CLEAN. Characteristics of the intervention and control arms of the trials are summarized in Table 2. The numbers of intention-to-treat (ITT) patients were 770 and 592 in the intervention and control arms, respectively. IV-tPA was administered equally in the intervention and control arms. Mechanical thrombectomy using stent retriever devices was performed in 302 (39%) patients of the intervention arm. IA-tPA was administered in 290 (38%) patients in the intervention arm. LVO was present in 526 (68%) and 462 (78%) patients in the intervention and control arms, respectively. General anesthesia was administered in 95 (28%) patients of the intervention arm.

Mean time from stroke onset to randomization was 204 min (IQR 152-251) and 223 min (IQR 170-312) in the intervention arms of MR CLEAN and REVASCAT, respectively. Mean time from stroke onset to randomization was 196 min (IQR 149-266) and 226 min (IQR 168-308) in the control arms of MR CLEAN and REVASCAT, respectively. IMS III did not report the mean time from stroke onset to randomization but required randomization within 40 minutes after the initiation of the tPA infusion.

### 3.3. Outcomes of Endovascular vs. Medical Management

**Figure 3:**
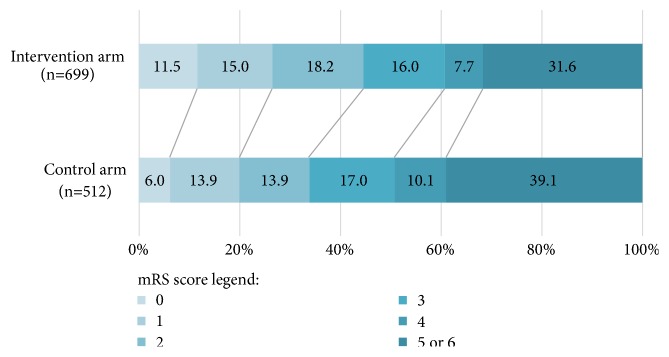
*Pooled modified Rankin Scale (mRS) scores at long-term follow-up*. Numbers represent percentages of patients in each outcome group. mRS range is 0-6: 0 indicating no symptoms, 1 no clinical disability, 2 slight disability, 3 moderate disability, 4 moderately severe disability, 5 severe disability, and 6 death. Percentages are rounded to the nearest whole number.

Pooled mRS score distribution at the end of long-term follow-up are shown in Figure 3. A Cochran-Mantel-Haenszel test demonstrated significant difference between the two arms (*p* = 0.0143). Scores of 5 and 6 were combined for the analysis. Of the 699 patients in the intervention arms with long-term follow-up assessment, 80 (11.5%), 105 (15%), 127 (18.2%), 112 (16%), 54 (7.7%), and 221 (31.6%) patients had mRS scores or 0, 1, 2, 3, 4, and 5, or 6, respectively. Long-term follow-up assessment was available in 512 patients of the control arms: 31 (6%), 71 (13.9%), 71 (13.9%), 87 (17%), 52 (10.1%), and 200 (39.1%) patients had mRS scores or 0, 1, 2, 3, 4, and 5, or 6, respectively. Functional independence (mRS score ≤ 2) at long-term follow-up favored endovascular stroke intervention (OR 1.51; 95% confidence interval [CI] 1.08–2.12;* p =* 0.02). There was no significant heterogeneity among the included trials in this analysis (*χ*^2^ = 3.63;* p *= 0.16; *I*^2^ = 45%). Functional independence (mRS score ≤ 2) at long-term follow-up was observed in 44.7% of patients in the endovascular group, compared with 33.8% in the control group.

**Figure 4:**
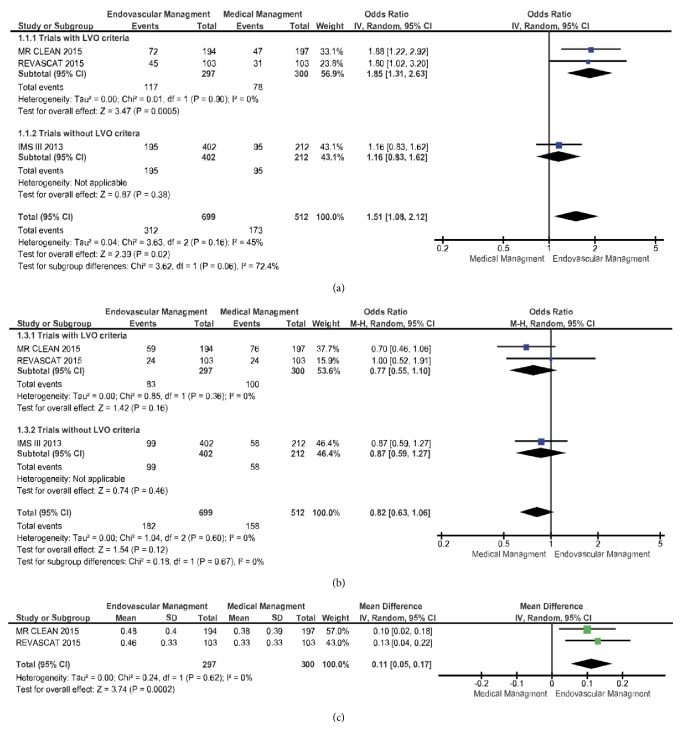
*Functional independence (mRS 0-2), mortality (mRS 6), and quality of life at long-term follow-up following endovascular or medical management of AIS due to LVO*. Forest plot of odds ratios (ORs) or mean difference for (a) functional independence (modified Rankin score or mRS 0-2), (b) all-cause mortality, (c) and quality of life (EQ-5D utility) at long-term follow-up. Estimated ORs and confidence intervals are shown, respectively, by the square box and horizontal line. Heterogeneity tests and effect size are shown.

No difference in mortality at long-term follow-up was demonstrated in the meta-analysis of pooled data (Figure 4) from the three studies (OR 0.82; 95% CI 0.63–1.06;* p* = 0.12).

### 3.4. Outcomes after Intervention vs. Medical Management Stratified by LVO Criteria

A sensitivity analysis was performed after excluding IMS III, evaluating only those patients with evidence of LVO on baseline neuroimaging (LVO criteria). MR CLEAN and REVASCAT demonstrated functional independence in favor of endovascular therapy (OR 1.85; 95% CI 1.31–2.63;* p* = 0.0005). IMS III alone, without LVO criteria, demonstrated no difference in functional independence between the 2 arms (OR 1.16; 95% CI 0.83–1.62;* p* = 0.38). There was no significant difference between the subgroups (*χ*^2^ = 3.62;* p* = 0.06; *I*^2^ = 72.4%).

Subgroup analysis of the two trials with LVO criteria demonstrated no difference in mortality between the two arms (OR 0.77; 95% CI 0.55– 1.10;* p* = 0.16). Similarly, IMS III, without LVO criteria, demonstrated no difference in mortality (OR 0.87; 95% CI 0.59–1.27;* p* = 0.46). No significant difference between the subgroups was found (*χ*^2^ = 0.18;* p* = 0.67; *I*^2^ = 0%).

Meta-analysis of pooled data from MR CLEAN and REVASCAT for quality of life (EQ-5D utility) is shown in Figure 4. There was a significant difference between the 2 arms in favor of endovascular therapy (Mean difference 0.11; 95% CI 0.05– 0.17;* p* = 0.0002). There was no significant heterogeneity among the included trials in this analysis (*χ*^2^ = 0.24;* p *= 0.62; *I*^2^ = 0%). The IMS III data on quality of life was unable to be pooled with the other two trials since the EQ-5D-3L score was transformed into quality adjusted days.

## 4. Discussion

Stroke patients often have significant disability, incurring a large societal monetary burden [30]. Despite multiple RCTs and meta-analyses highlighting the safety and efficacy of endovascular thrombectomy at a standard 3-month period, the long-term benefits remain unclear. In previous meta-analyses published by Goyal et al.* 2016* and Tsivgoulis et al.* 2016*, successful revascularization rates as well as functional independence at a 3-month follow-up period were shown using pooled data from the ESCAPE, EXTEND IA, MR CLEAN, REVASCAT, and SWIFT PRIME trials [24, 31]. In contrast, our meta-analysis pooled data from the REVASCAT, MR CLEAN, and IMS III trials at their respective long-term follow-up time points. We show that endovascular intervention coupled with medical therapy significantly improves long-term functional independence and quality of life (QOL) in patients with AIS. Quality of life was not reported in previous meta-analyses as it was not reported in most trials with short-term follow-up. Despite a lower long-term mortality rate for the endovascularly managed patients (26% vs. control 31%), this was not statistically significant.

All included studies were quantitatively assessed for heterogeneity and none was observed. Despite this, it is important to note that data obtained from the IMS III trial is qualitatively heterogenous from the other two trials. IMS III did not have LVO inclusion criteria and most of the endovascular interventions performed were without stent retriever devices. However, despite these limitations, long-term follow-up of patients in the IMS III trial demonstrated a significant benefit in the functional outcomes and quality of life in patients with severe stroke (NIHSS ≥ 20) who received endovascular therapy as compared to standard medical treatment alone.

The pooled data from the three trials demonstrates that when compared to standard medical treatment, endovascular treatment increases the odds for functional independence at long-term follow-up by over 50% (OR 1.51; 95% CI 1.08-2.12;* p* = 0.02) in patients with AIS. More patients demonstrated functional independence at long-term follow-up in the endovascular arm than the control arm (44.6% vs. 33.7%, respectively); similar results were also observed at short-term follow-up. Furthermore, when including only trials with LVO inclusion criteria (i.e., REVASCAT and MR CLEAN) functional independence with endovascular treatment nearly doubles (OR 1.85; 95% CI 1.31-2.63;* p* = 0.0005). In LVO inclusion trials, pooled long-term functional independence seen with endovascular management is similar to the pooled 90-day benefits reported by Goyal et al. (OR 2.35,* p* < 0.0001) [24]. The slight discrepancy observed between the long-term and 90-day odds ratios, OR 1.85 vs. 2.35, is likely secondary to the fact that the pooled 90-day data included more studies (MR CLEAN, ESCAPE, REVASCAT, SWIFT PRIME, and EXTEND IA), two of which, EXTEND IA and SWIFT PRIME, required perfusion selection criteria and reported higher functional independence rates than the other studies. Furthermore, these studies do not have long-term follow-up data available.

There has been some uncertainty in stroke literature regarding the optimal time point to assess follow-up mRS scores [32]. The majority of RCTs have utilized the 90-day time point based on the premise that the majority of mRS change, both negative and positive, are observed within the first three months following stroke [32]. There is some evidence to reduce the follow-up time point for thrombectomy trials. REVASCAT's data show that their 90-day outcomes were predictive of their 12-month outcomes (proportion of treatment effect 0.89); furthermore, their 5-day time point was also predictive of their 12-month outcomes (proportion of treatment effect 0.80) [29]. On the other hand, 12-month outcomes from the IMS III demonstrate a significant benefit for endovascular intervention in patients suffering a severe stroke (NIHSS ≥ 20), a benefit that was not previously detected with their 90-day timepoint, suggesting that a longer follow-up time should be considered [27]. However, REVASCAT investigators considered these IMS III results and in a post hoc analysis failed to find any relationship between stroke severity and outcomes, at either short-term or long-term follow-up [29]. These trial-to-trial differences in long-term outcome results are likely a result of LVO inclusion heterogeneity. Our results in this meta-analysis demonstrate that despite a small decrease in the unadjusted odds ratio from short-term to long-term follow-up, the documented short-term beneficial effects of endovascular therapy likely predict the long-term outcomes.

One argument for longer follow-up is to ensure that the benefits, and thus the cost-effectiveness, of thrombectomy for stroke are maintained. Amongst the trials with LVO inclusion criteria we noticed a different trend in patients with excellent outcomes (mRS 0 or 1) between their respective 90-day and long-term time points. In MR CLEAN, there were less patients with excellent functional outcomes at the two-year follow-up compared to the 90-day time point whereas in REVASCAT there was an increase of patients with excellent outcomes at the longer time point. MR CLEAN investigators suggested that the initial poststroke physical therapy may mask a portion of the deficits, explaining the long-term decrease in excellent outcomes. This justification is unlikely since the opposite pattern is evident in the REVASCAT trial. Perhaps most of the functional decline occurred in the second year following stroke, a timepoint studied in MR CLEAN but not REVASCAT. MR CLEAN also had no ischemic core exclusion criteria whereas REVASCAT excluded patients with a large ischemic core as determined by ASPECT score. It would be interesting to evaluate the REVASCAT cohort for an additional year to observe how the proportion of excellent outcome patients varies from one year to two years after thrombectomy.

Improvements in patient-centered outcomes including mobility, self-care, usual activities, pain or discomfort, and anxiety or depression provide further evidence regarding the effectiveness of mechanical thrombectomy. At the 90-day time point, REVASCAT investigators reported quality of life improvements with mechanical thrombectomy whereas MR CLEAN investigators failed to find significant improvement [18, 33]. Patients who suffered AIS from LVO in the endovascular group reported a better health-related quality of life, via EQ-5D questionnaire, than those in the control arm at long-term follow-up. This suggests that a longer timepoint may be required to discern the quality of life differences in stroke trials, especially in those trials that do not exclude patients based on ischemic core size.

Though not included in the quantitative analysis, Lopez-Cancio et al. performed evaluations of cognitive function using the Trail Making Test (TMT) for the REVASCAT cohort at 3 months and 12 months [34]. Among functionally independent patients, those in the thrombectomy treatment arm completed tasks quicker and with less error in the TMT neuropsychological test than those in the control group, suggesting better attention, visuospatial abilities, and cognitive flexibility. However, these cognitive benefits were not observed, at either 90-day or 12-month follow-up, in patients who were functionally dependent after stroke (mRS > 2), suggesting diminished cognitive benefits of mechanical thrombectomy in patients who do not reach postintervention functional independence. This highlights the necessity for improving factors that lead to higher postthrombectomy functional independence such as emergency prenotification, time to revascularization, and rates of TICI 3 recanalization. As with functional outcome and health-related quality of life, Lopez-Cancio et al. observed an inverse relationship between infarct volume and executive function. These results also support the use of cognitive function in addition to mRS for outcome assessment in future stroke trials.

### 4.1. Limitations

The included trials are not without limitations. REVASCAT and MR CLEAN were terminated early. In all studies, the practitioners and patients were unblinded to treatment modality.

Our meta-analysis is limited by the pooled data available from the included studies, a common limitation among meta-analyses. Additionally, included studies are relatively outdated with regard to mechanical thrombectomy devices. Furthermore, findings are not necessarily generalizable to the distal anterior circulation or the posterior circulation since the LVO inclusion criteria was limited to the anterior circulation.

## 5. Conclusions

In this meta-analysis we pooled data from REVASCAT, MR CLEAN, and IMS III trials comparing mechanical thrombectomy to standard medical management with IV-tPA alone. We demonstrate that, compared to medical management, endovascular therapy results in favorable functional independence, health-related quality of life, and cognitive function at long-term follow-up for patients with AIS. Furthermore, the standard 90-day timepoint offers a fair representation of the long-term outcomes.

## Figures and Tables

**Table 1:** Included trials and their respective study designs.

	Trial	IMS III	MR CLEAN	REVASCAT
Enrollment Criteria	Publication year	2013	2015	2015
	Time period	2006-2012	2010-2014	2012-2014
	Location	North America, Europe, Australia	Netherlands	Spain
	No. of Centers	58	16	4
	No. of Patients	656	500	206
	Last known well to randomization, h	≤5	≤6	≤8
	Age, y	≥18^a^	≥18	18-85^c^
	NIHSS score	≥10, or ≥8 with LVO^b^	≥2	≥6
	LVO	NA^a^	ICA, MCA (M1/ M2) ACA (A1/ A2)	ICA, MCA (M1)
	ASPECTS	NA^b^	NA	≥7, CT; ≥6, MRI
	Endovascular intervention	IA thrombectomy, IA-tPA, IV-tPA	IA thrombectomy, IA-tPA, IV-tPA	IA thrombectomy, IV-tPA
	Control arm	IV-tPA	IV-tPA	Standard therapy

	Primary endpoint	mRS ≤2 at 1 y	mRS at 2 y	mRS at 1 y

	Follow-up duration	1 y	2 y	1 y

ACA, anterior cerebral artery; ASPECTS, Alberta Stroke Program Early CT score; CTA, CT angiography; d, days; IA, intra-arterial; ICA, internal carotid artery; LVO, large vessel occlusion; MCA, middle cerebral artery; mRS, modified Rankin Scale; NA, not applicable; No., number; tPA, tissue plasminogen activator

^a^ After 284 patients enrolled, protocol altered to no upper limit for age, identification of occlusion with CTA was allowed for patients with NIHSS score of 8 or 9

^b^ ASPECTS <4 used as guideline when evaluating >1/3 region of territory involvement, but not exclusion criteria.

^c^ After enrollment of 160 patients, inclusion criterion was changed from 80 years old to up to 85 years old with >8 ASPECTS.

**Table 2:** Characteristics of intervention and control arms of included studies.

	Trial	IMS III	MR CLEAN	REVASCAT	Total
Intervention Arm	ITT patients, n	434	233	103	*770*
	Stent retrieve device, n (%)	14 (3)	190 (82)	98 (95)	*302 (39)*
	IA-tPA, n (%)	266 (61)	24 (10)	0 (0)	*290 (38)*
	IV-tPA, n (%)	434 (100)	203 (87)	70 (68)	*707 (92)*
	Mean/median NIHSS score	17	17	17	
	Mean/median ASPECTS	NA	9	7	
	Mean/median age, y	69	65.8	65.7	
	LVO, n (%)	190 (44)^a^	233 (100)	103 (100)	*526 (68)*
	GA, n (%)	NR	88 (38)	7 (7)	*95 (28)*
	Mean/median time from onset to groin puncture, min	208	260	269	
	Median time from onset to randomization, min (IQR)	NR^b^	204 (152-251)	223 (170-312)	

Control Arm	ITT patients, n	222	267	103	*592*
	IV-tPA, n (%)	222 (100)	242 (91)	80 (78)	*544 (92)*
	Mean/median NIHSS score	16	18	17	
	Mean/median ASPECTS	NA	9	8	
	Mean/median age, y	68	65.7	67.2	
	LVO, n (%)	92 (41)^a^	267 (100)	103 (100)	*462 (78)*
	Mean/median time to tPA, min	121.2	87	105	
	Median time from onset to randomization, min (IQR)	NR^b^	196 (149-266)	226 (168-308)	

ASPECTS is Alberta Stroke Program Early CT score; GA is general anesthesia; IA is intra-arterial; ITT is intention-to-treat; LVO is large vessel occlusion; NA is not applicable; NR is not reported; tPA is tissue plasminogen activator.

^a^ After 284 patients had undergone randomization, identification of occlusion with CT angiography could determine trial eligibility for patients with NIHSS score of 8 or 9.

^b^ Randomization was required within 40 minutes after the initiation of the tPA infusion.

## Supplementary Materials

Supplementary Materials The supplemental file attached is the prisma checklist for the meta-analysis that was submitted.
